# Functional Roles of Astrocyte Calcium Elevations: From Synapses to Behavior

**DOI:** 10.3389/fncel.2017.00427

**Published:** 2018-01-17

**Authors:** Sónia Guerra-Gomes, Nuno Sousa, Luísa Pinto, João F. Oliveira

**Affiliations:** ^1^Life and Health Sciences Research Institute (ICVS), School of Medicine, University of Minho, Braga, Portugal; ^2^ICVS/3B’s – PT Government Associate Laboratory, Braga, Portugal; ^3^DIGARC, Polytechnic Institute of Cávado and Ave, Barcelos, Portugal

**Keywords:** astrocyte, calcium, synapse, neural circuit, behavior

## Abstract

Astrocytes are fundamental players in the regulation of synaptic transmission and plasticity. They display unique morphological and phenotypical features that allow to monitor and to dynamically respond to changes. One of the hallmarks of the astrocytic response is the generation of calcium elevations, which further affect downstream cellular processes. Technical advances in the field have allowed to spatially and to temporally quantify and qualify these elevations. However, the impact on brain function remains poorly understood. In this review, we discuss evidences of the functional impact of heterogeneous astrocytic calcium events in several brain regions, and their consequences in synapses, circuits, and behavior.

## Different Forms of Calcium Elevations in Astrocytes: the Complexity of Simplicity

Astrocytes have emerged as key players in the regulation of synaptic physiology. They display complex and heterogeneous morphological structures and are able to sense and respond to environmental signals, modulate neuronal activity, synaptic transmission, and vascular function (Araque et al., 2014; Perea et al., 2014; Petrelli and Bezzi, 2016); for review (Petzold and Murthy, 2011; Zorec et al., 2012). Synaptic activity is integrated by astrocytes (Perea and Araque, 2005) and might result in intracellular calcium (Ca^2+^) elevations with specific spatial and temporal properties. These might appear as “global” and/or “focal” responses within the astrocytic complex morphology. It is yet unclear if global Ca^2+^ elevations are more representative of an integrative response or if they are the result of a linear summation of Ca^2+^ fluctuations or inositol 1,4,5-trisphosphate (IP_3_) levels; nevertheless, these have been used for over two decades as a readout of astrocytic function (Khakh and McCarthy, 2015); for review (Rusakov et al., 2014; Volterra et al., 2014). Ca^2+^ elevations may appear spontaneously or may be triggered by endogenous or exogenous stimuli. Traditionally, the use of Ca^2+^ indicator dyes allowed for the detection of Ca^2+^ elevations within astrocyte somata, but failed to efficiently track them in the fine processes (Wang et al., 2006; Takata and Hirase, 2008; Nizar et al., 2013). This hindered the complete interpretation of Ca^2+^ dynamics. More refined methods to image astrocyte Ca^2+^ signals have been recently described (Kanemaru et al., 2014; Srinivasan et al., 2016; Bindocci et al., 2017); for review (Shigetomi et al., 2016).

There are several mechanisms that trigger the elevation of astrocyte intracellular Ca^2+^ levels. The activation of G_q_-protein-coupled receptors (GPCR) triggers the IP_3_ signaling cascade and results in robust intracellular Ca^2+^ elevations, mainly via the IP_3_ receptor type 2 activation (IP_3_R2) (Petravicz et al., 2008; Takata et al., 2011; Navarrete et al., 2012); for review (Parpura et al., 2011). Curiously, GABA_B_ receptor (G_i_-coupled GPCRs) activation was also shown to trigger intracellular Ca^2+^ elevations in astrocytes (Serrano et al., 2006; Mariotti et al., 2016; Perea et al., 2016). Moreover, astrocytes express several types of transient receptor potential (TRP) channels (Verkhratsky et al., 2013). The spontaneous opening of TRPA1 channels was shown to contribute to basal Ca^2+^ levels and to a fraction of intrinsic fluctuations (Shigetomi et al., 2012, 2013), and their blockade contributes to a slight decrease in resting Ca^2+^ levels (Agarwal et al., 2017). Additionally, TRPC channels modulate Ca^2+^-dependent vesicular glutamate release in cortical astrocytes by contributing to the generation of store-operated Ca^2+^ entry (SOCE) (Reyes et al., 2013). More recently, Agarwal et al. (2017) reported that mitochondria, which are abundant in astrocytic processes, are the main active source of Ca^2+^ for localized events in far distant microdomains.

While our understanding of the origin and features of astrocytic Ca^2+^ signaling is steadily growing, its functional consequences to the surrounding cellular circuits remain poorly understood. Therefore, this review will focus on the available data showing functional outputs of astrocytic Ca^2+^ elevations. The reader may find useful reviews and discussions elsewhere on the origin and features of astrocyte Ca^2+^ elevations (Scemes and Giaume, 2006; Agulhon et al., 2008; Leybaert and Sanderson, 2012; Volterra et al., 2014; Khakh and McCarthy, 2015; Bazargani and Attwell, 2016; Shigetomi et al., 2016), and on the role of astrocyte Ca^2+^ in pathology (Nedergaard et al., 2010; Agulhon et al., 2012; Vardjan et al., 2017).

## Functional Roles of Astrocyte Calcium Elevations

The advent of novel techniques and approaches allowed for the quantification of astrocytic Ca^2+^ elevations and the characterization of their spatiotemporal dynamics in several brain regions. Below, we will discuss relevant studies that report on the functional influence of astrocytic Ca^2+^ elevations on synapses, circuits, and behavior.

### Astrocyte Calcium and Synapses

**FIGURE 1:**
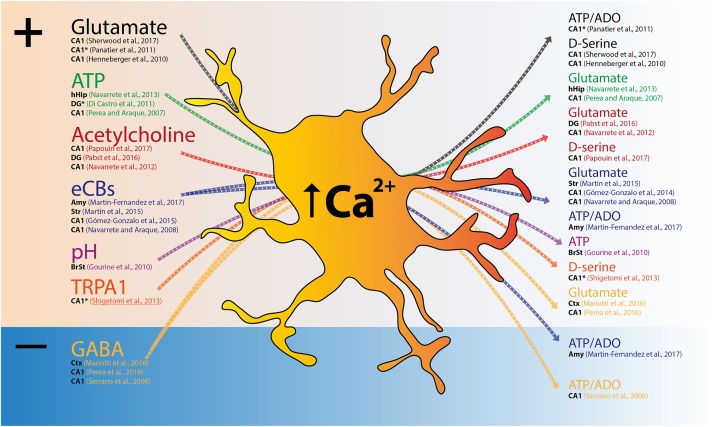
Excitatory and/or inhibitory signals trigger Ca^2+^ elevations in astrocytes and lead to gliotransmitter release. Scheme depicting input signals that trigger astrocyte Ca^2+^ elevations and respective transmitter release. Both excitatory (+) and inhibitory (–) signals cause global or focal Ca^2+^ elevations in astrocytes (Left), and precede gliotransmitter release that might exert excitation or inhibition of neighboring synapses (Right). For each reference, the region studied is indicated in black (Amy, Amygdala; BrSt, brainstem; CA1, CA1 subfield of the hippocampus; Ctx, cortex; DG, dentate gyrus; hHip, human hippocampus; Str, Striatum). ^∗^Indicates the studies that described functional consequences to focal Ca^2+^, rather than global Ca^2+^ responses.

The studies reviewed in this section describe experiments performed in rat and mouse brain slices. Several pieces of evidence indicate that astrocytic Ca^2+^ elevations precede the release of gliotransmitters, which may ultimately modulate synaptic transmission (**Figure 1**). Different levels of neuronal activity appear to scale astrocytic Ca^2+^ levels, leading to orchestrated (multi)synaptic responses (for review, Araque et al., 2014). In the hippocampus, ATP-evoked astrocyte Ca^2+^ was shown to promote glutamate release, which consequently facilitates synaptic transmission through the activation of mGluRs (Perea and Araque, 2007). Although further attempts to control astrocytic Ca^2+^ signaling failed either to affect general readouts of synaptic transmission and plasticity in the hippocampus [using MrgA1 and IP_3_R2 KO mouse models (Petravicz et al., 2008; Agulhon et al., 2010)] or to detect glutamate release in the hippocampus or striatum [upon Designer Receptor Exclusively Activated by Designer Drugs (DREADD) activation (Chai et al., 2017)], a series of studies have supported this hypothesis. In fact, the tight modulation of astrocytic intracellular Ca^2+^ is crucial for D-serine-dependent long-term potentiation (LTP) of hippocampal CA1 pyramidal cells (Henneberger et al., 2010). Accordingly, a recent study has showed that acute blockade of Ca^2+^-dependent IP_3_ mechanisms impairs LTP, but that this can be rescued by exogenous D-serine (Sherwood et al., 2017).

Astrocytic Ca^2+^ elevations appear to also promote alternative forms of plasticity in the brain. In the hippocampus, cholinergic afferents from the medial septum modulate CA1 synaptic plasticity via an astrocytic Ca^2+^-dependent mechanism that triggers glutamate release and consequent activation of mGluRs in neurons (Navarrete et al., 2012). This mechanism of synaptic modulation is not restricted to the hippocampus since astrocytic Ca^2+^ elevations also mediate muscarinic acetylcholine receptor-dependent plasticity in the somatosensory cortex (Takata et al., 2011), as it will be discussed in the next chapter. Moreover, a recent study showed that the cholinergic input to astrocytes, driven by active/sleep phases, controls Ca^2+^-dependent D-serine release, ultimately gearing up NMDA receptor activation at CA1 synapses (Papouin et al., 2017). In a different hippocampal sub-field, the cholinergic activation of hilar astrocytes triggers intracellular Ca^2+^ elevations that precede the activation of hilar inhibitory interneurons, causing a long-lasting GABAergic inhibition of dentate granule cells (Pabst et al., 2016). Further evidences point out that astrocytic Ca^2+^ mediates endocannabinoid-dependent plasticity. Navarrete and Araque (2008) reported that the activation of astrocytic cannabinoid type 1 (CB1) receptors leads to intracellular Ca^2+^ elevations, triggering glutamate release and slow inward currents (SICs) in vicinal pyramidal neurons. Furthermore, endocannabinoid-triggered elevation of astrocytic Ca^2+^ levels contributes to heteroneuronal LTP (Gómez-Gonzalo et al., 2015). In the striatum, a circuit-specific astrocyte-neuron signaling takes place in which the release of endocannabinoids promotes Ca^2+^ elevations in a specific population of astrocytes, triggering the release of glutamate that modulates excitability and synaptic transmission (Martín et al., 2015). Finally, in the medial central amygdala (CeM), astrocytes receive endocannabinoid signaling to release ATP and depress excitatory synapses from basolateral amygdala via A_1_ adenosine receptor activation, and enhance inhibitory synapses from the lateral subdivision of the central amygdala via A_2A_ receptor activation. This redundancy system results in CeM neuronal inhibition with impact in animal behavior (Martin-Fernandez et al., 2017).

Interestingly, astrocytic Ca^2+^ elevations are not exclusive to the action of classic excitatory transmitters. Activation of GABA receptors in astrocytes was shown by different research groups to trigger Ca^2+^ elevations with functional synaptic consequences. Specifically, GABAergic heterosynaptic depression in the hippocampal CA1 subfield requires astrocytic Ca^2+^ elevations and ATP release, whose metabolite adenosine will activate A_1_ receptors (Serrano et al., 2006). More recently, Mariotti et al. (2016) showed that the activation of GABA_B_ receptors evoked somatic Ca^2+^ elevations, consequently leading to the occurrence of SICs in cortical pyramidal neurons. Curiously, these effects were absent in IP_3_R2 KO mice suggesting a relationship between G_i_-coupled GPCRs and IP_3_ signaling in astrocytes. Moreover, the activation of astrocytic GABA_B_ receptors triggered intracellular Ca^2+^ elevations in hippocampal CA1 astrocytes, which led to synaptic potentiation via glutamate release and modulation of presynaptic group I mGluRs (Perea et al., 2016).

Astrocytic Ca^2+^ signaling also plays a role in structural integrity of synapses. It was reported that the IP_3_ “sponge” mouse model, whose astrocytic IP_3_ signaling is impaired, has a reduced astrocytic coverage of asymmetric synapses leading to modulation of glutamatergic transmission (Tanaka et al., 2013). In accordance, activity-related structural remodeling of astrocytic processes in the vicinity of synapses was shown to depend on presynaptic activity and to require G-protein-mediated Ca^2+^ elevations in astrocytes, both in the hippocampus and in the cortex (Perez-Alvarez et al., 2014).

Finally, Navarrete et al. (2013) studied cortical and hippocampal human brain samples and showed that increases in intracellular Ca^2+^ levels of both cortical and hippocampal astrocytes are accompanied by an increase in SICs frequency. These observations confirm that, partially at least, astrocyte Ca^2+^ elevations are also required to modulate synapses in the human brain.

Most of the studies mentioned above regarding synaptic modulation reported consequences of somatic (or in major processes) Ca^2+^ elevations. Recently, several elegant studies suggested that Ca^2+^ signals occurring only at astrocyte microdomains are sufficient to modulate synaptic events. Specifically, Ca^2+^ signals in astrocyte processes of the hippocampal dentate gyrus are relevant for basal synaptic function, since the application of a Ca^2+^ chelator or an antagonist for a GPCR predominantly expressed in astrocyte processes decreased synaptic efficacy (Di Castro et al., 2011). Similarly, mGluR-dependent astrocytic Ca^2+^ signaling was shown to mediate the regulation of basal transmission in CA1 pyramidal neuron synapses (Panatier et al., 2011). The Ca^2+^ signaling that occurs at the distant processes appears to originate from distinct sources, other than the endoplasmic reticulum. For instance, TRPA1 channels mediate a transmembrane Ca^2+^ flux pathway with contributions to basal Ca^2+^ levels and regulation of interneuron inhibitory synapse efficacy via GAT-3 (Shigetomi et al., 2012). In addition, the same group showed that TRPA1 activation contributes to the release of D-serine into the extracellular space, which consequently influences NMDA-dependent hippocampal plasticity (Shigetomi et al., 2013).

Most of the available data resulted from experimental approaches that block general astrocyte Ca^2+^ elevations, namely by using Ca^2+^ chelators or genetic IP_3_R2 deletion. This may explain the current bias toward effects of global (soma and main processes) Ca^2+^ signals rather than that of focal events (restricted to cellular microdomains). Nevertheless, it is now clear that global and focal signals coexist in specific spatio-temporal maps. Despite the 10- to 100-fold scale that distinguishes global and focal signals, both have been shown to precede transmitter release and modulate different forms of synaptic transmission (for review, Volterra et al., 2014). Future studies are needed to further discriminate their functional consequences.

Altogether, these sets of data suggest that astrocytes integrate brain circuits following two physiological mechanisms (**Figure 1**). First, the activation of astrocytes appears to represent a novel integration mechanism. Astrocyte Ca^2+^ elevations not only occur upon activation through various excitatory transmitters (e.g., glutamate, ATP, or acetylcholine), but can also be triggered by the inhibitory transmitter GABA. This means that, independently of the nature of the transmitter, astrocytes will be excited/activated (**Figure 1**, left), suggesting they integrate some brain circuits as a redundant layer that reads excitatory and inhibitory neuronal inputs similarly. This concept may add up to the mechanisms of coincidence detection proposed a decade ago by Perea and Araque (2005, 2007). Second, upon activation and Ca^2+^ elevation, astrocytes release gliotransmitters that ultimately cause either neuronal excitation (e.g., glutamate or D-serine) or inhibition (e.g., ATP degraded to adenosine). Curiously, the available literature indicates that astrocytes provide, in most cases, an excitatory output. Nevertheless, astrocytes also release ATP, which is readily degraded to adenosine and in turn activates A_1_ receptor leading to synaptic inhibition (Serrano et al., 2006; Martin-Fernandez et al., 2017). This suggests that the type of modulation performed by astrocytes relies not only on the type of transmitter released (which is an intrinsic specificity of the astrocyte), but also on the nature of the receptors expressed by the neighboring cells (which depends on the nature of the neural circuit). In accordance, Martin-Fernandez et al. (2017) also showed that astrocyte-derived ATP results ultimately in excitation or inhibition, depending on the type of receptors found by adenosine. Whether brain circuits are endowed with exclusively excitatory and/or exclusively inhibitory astrocytes, or if these cells play both roles simultaneously (i.e., express machinery to produce, load, and release different transmitters), is still unknown. The first case would not be surprising, since it is the reality for the different neuronal cells (e.g., excitatory glutamatergic vs. inhibitory GABAergic). While recent technical advances have provided fruitful reports of unexpected physiological processes, future studies are needed to confirm their relevance for circuit function.

### Astrocyte Calcium and Neural Circuits

The difficulty to measure and/or manipulate intracellular astrocytic Ca^2+^ in the intact brain may explain the lack of studies that report on its functional consequences for circuit and behavior computation. Still, recent reports indicate that the Ca^2+^-dependent modulation of single synapses (as reviewed above) also have an expected impact on brain circuits.

Regarding the control of cortical synchronization, it was observed that astrocyte Ca^2+^ elevations *in vivo* regulate extracellular glutamate levels, which consequently triggers a slow neuronal rhythm in the brain. This event is characterized by synchronized neuronal firing across different behavioral states, and points to an important regulatory role of astrocytes in cortical circuits (Poskanzer and Yuste, 2016). This observation is in line with previous studies performed in brain slices of the cortex and hippocampus that showed reduced neural synchrony upon disruption of astrocytic Ca^2+^ elevations (Poskanzer and Yuste, 2011; Sasaki et al., 2014). Neural rhythmicity in these regions is essential for several behavioral functions such as attention, learning, memory, and control of sleep/wake cycles (for review, Oliveira et al., 2015). IP_3_R2-dependent signaling appears to support ripple-type events that occur during non-theta periods in the CA1 of rodents, a mechanism that might be related to the emotional consequences of social isolation (Tanaka et al., 2017).

In the rat trigeminal sensorimotor circuit for mastication, neural rhythmicity arises after sensory encoding. In this circuit, astrocytes actively respond to sensory stimuli by elevating their intracellular Ca^2+^ levels and, thus, regulating neuronal rhythmic activity, in brainstem slices (Morquette et al., 2015). Furthermore, in thalamo-cortical circuits, astrocytic Ca^2+^ elevations contributed to the modulation of sensory transmission. More specifically, the activation of mGluR2 triggers intracellular Ca^2+^ elevations in astrocytes, which are blocked by the astrocyte-specific toxin fluorocitrate. This effect described *in vivo* and in brain slices is linked to sensory inhibition in the rodent thalamus (Copeland et al., 2017). Furthermore, simultaneous stimulation of whiskers and the nucleus basalis of Meynert revealed that astrocytic Ca^2+^ elevations precede muscarinic acetylcholine receptor (mAChR)-dependent plasticity in the somatosensory cortex *in vivo*. This process is dependent on IP_3_R2 signaling and extracellular D-serine (Takata et al., 2011). In the visual cortex, IP_3_R2-mediated astrocytic Ca^2+^ elevations are also critical for the integration of visual sensory inputs with the nucleus basalis afferent information (Chen et al., 2012). Moreover, transcranial direct current stimulation (tDCS) was shown to enhance sensory-evoked cortical responses, through elevation of astrocytic intracellular Ca^2+^ via IP_3_R2 (Monai et al., 2016). Thus, the authors propose that astrocytic Ca^2+^ elevations might mediate, at least in part, the recognized improvements obtained by tDCS in neuropsychiatric and neurological conditions. Finally, astrocyte Ca^2+^ elevation appears to also be involved in the maintenance of homeostatic mechanisms. Astrocytes in brainstem chemoreceptor areas respond to physiological decreases in pH with vigorous elevations in intracellular Ca^2+^. These were shown to trigger the release of ATP, inducing adaptive increases in breathing (Gourine et al., 2010).

Astrocyte Ca^2+^ elevations appear to control the function of brain circuits at least in two different forms. On one hand, astrocytes appear to control plasticity in synapses occurring between neurons projecting to long distances, similarly to the control of local synapses within one brain region, as discussed above. On the other hand, astrocytes of a specific region appear to support local neural synchronization states, controlling the circuit output, most likely in a multi-synaptic process. Whether the two forms of modulation use similar mechanisms, i.e., whether regional integration is a product of 10 to 1000 of synapses being similarly integrated, is still unknown. However, it seems that both temporal and spatial properties of Ca^2+^ signals are important for this modulation to occur.

### Astrocyte Calcium Effects on Behavior

The study of rodent models that display altered astrocyte function indicated that astrocytes play important roles in the production of behavior outputs in different dimensions (cognition, emotion, motor, and sensory processing) (for review, Oliveira et al., 2015). As reviewed above, astrocytic Ca^2+^ signaling is reported to mediate synaptic modulation in different brain circuits. Genetic interference in this astrocytic hallmark has been the main strategy used to assess the role played by astrocyte Ca^2+^ elevations in behavior, with different laboratories using mostly two approaches: (1) triggering intracellular Ca^2+^ elevations via chemogenetic activation of GPCR signaling, and (2) inhibiting IP_3_ signaling by deletion of IP_3_ receptors or by buffering IP_3_.

The genetic deletion of IP_3_R2, specifically in about 80% of glial fibrillary acidic protein (GFAP)-positive cells (at least in the cortex, hippocampus, and substantia nigra), does not appear to influence spatial memory (Petravicz et al., 2014). However, the attenuation of IP_3_ signaling in GLT1-positive cells led to partial cognitive impairment (Tanaka et al., 2013). Despite the extensive evidence showing a clear influence of astrocyte Ca^2+^ events on synapses and circuits involved in cognitive behavior (e.g., hippocampus), these two studies provided only modest evidence to support it. We believe that further studies using more specific tools to modulate astrocyte Ca^2+^ will reveal additional links to cognition. Indeed, we recently showed that spatial learning and memory rely on astrocyte exocytosis, which is a Ca^2+^-dependent mechanism, and therefore might be dependent on astrocyte integration of surrounding activity (Sardinha et al., 2017).

IP_3_-dependent astrocytic Ca^2+^ signaling does not seem to be related to anxiety-like behavior as indicated by studies using a constitutive (Cao et al., 2013; Tanaka et al., 2013) or conditional (Petravicz et al., 2014) IP_3_R2 deletion. Regarding the depressive-like behavior, the available data is not consistent. While the constitutive deletion of IP_3_R2 was shown to trigger some forms of a depressive phenotype (Cao et al., 2013), these are completely absent in the model with conditional deletion (Petravicz et al., 2014). Curiously, the GFAP-MrgA1 model that allows stimulation of astrocyte Ca^2+^ displayed decreased learned helplessness in the forced swim test (Cao et al., 2013). Further experimentation is required to clarify these apparently contradictory results. Finally, a recent study indicated that the astrocytic control of excitatory/inhibitory inputs to the central amygdala is crucial for fear-related behavior (Martin-Fernandez et al., 2017).

Regarding motor function, the activation of IP_3_ “sponge” or deletion of IP_3_R2 does not seem to interfere with exploratory behavior (Cao et al., 2013; Tanaka et al., 2013; Petravicz et al., 2014). Interestingly, the chemogenetic activation of astrocyte G_q_-coupled signaling led to an impairment in motor coordination in an IP_3_R2-independent manner (Agulhon et al., 2013). The specific ablation of IP_3_R2 in GLAST-positive cells resulted in an impairment in motor-skill learning of a forelimb reaching task (Padmashri et al., 2015). More so, the attenuation of IP_3_/Ca^2+^ signaling in astrocytes resulted in modulation of the rodent sleep, by increasing the time spent in the rapid eye movement (REM) phase and the frequency of REM periods (Foley et al., 2017).

While the research carried out so far has provided us with critical knowledge of the influence of astrocytic Ca^2+^ in synaptic or circuit function, further research should be performed to complement the rather sparse evidence on the influence of astrocytes on behavior. Moreover, further evidence will help clarify the discrepant results and reconcile the existent observations. For instance, the different observed cognitive outcomes might be related to the different mechanism used to target intracellular Ca^2+^ (full deletion of IP_3_R2 vs. IP_3_ “sponge”), or by the promotor used to drive astrocyte specificity (GFAP vs. GLT1) that could result in differential regional expression. Moreover, there are at least five different IP_3_R2 KO strains with different genetic backgrounds (Oliveira et al., 2015); this might not be critically relevant for studies of synaptic plasticity in brain slices, but it may easily lead to distinct observations in behavior tests. Additionally, the different studies should be conducted in a standardized form to allow the linear comparison of the results.

All rodent models reported above result on drastic modulation of global Ca^2+^ elevations. Intracellular Ca^2+^ rises may trigger a multitude of signaling pathways. Thus, it must always be considered that the interference with global Ca^2+^ events may trigger several simultaneous consequences, which might lead to confounding results. Although these models might be very useful when carefully assessed, the research field requires the development of more specific models to allow temporal and, more importantly, local control of Ca^2+^ signals in physiological processes.

## Conclusion

Intracellular Ca^2+^ elevations are the mechanism by which astrocytes decode afferent information and generate functional outputs. Here, we discussed relevant information showing that astrocytic Ca^2+^ elevations have a direct impact on synapses (gliotransmitters release, synaptic plasticity, and integrity) and influence circuit outputs (sensory plasticity and network synchronization). However, the behavior readouts of rodents whose global astrocytic Ca^2+^ was manipulated remain inconsistent.

Astrocytic Ca^2+^ signals exhibit critical spatio-temporal features that need to be fully understood to allow for the careful assessment of their functional implications. Among them: spontaneous vs. evoked events; fast vs. slow elevations; synchronous vs. asynchronous; global (soma and main processes) vs. focal (microdomain); single vs. multiple astrocyte; local vs. across brain regions; IP_3_-dependent vs. IP_3_-independent mechanisms. The complementary application of existing tools, as well as the development of accurate models to tackle its unique and heterogeneous features, will provide us with novel insights on its actions and consequences.

## Author Contributions

All authors listed have made a substantial, direct and intellectual contribution to the work, and approved it for publication.

## Conflict of Interest Statement

The authors declare that the research was conducted in the absence of any commercial or financial relationships that could be construed as a potential conflict of interest.
